# Editorial to the Special Issue “Theranostic Drug Delivery: Prospects and Problems”

**DOI:** 10.3390/biomedicines12071533

**Published:** 2024-07-10

**Authors:** M. R. Mozafari

**Affiliations:** Australasian Nanoscience and Nanotechnology Initiative (ANNI), 8031 Monash University LPO, Clayton, VIC 3168, Australia; dr.m.r.mozafari@gmail.com

The technical phrase theragnostic (also known as theranostic) was first introduced to the scientific community in the year 1998 by John Funkhouser, to describe a methodology or procedure employed to achieve disease diagnosis and treatment simultaneously [1]. Some scholars, however, have added a third element and defined the terminology as the combined diagnosis, cure and follow up of an ailment [2]. Currently, theragnostic/theranostic has become an advanced research area that is considered as one of the most promising precision medicine techniques, with particular applications in cancer detection and cure. This novel protocol exploits cancer-specific receptors located on the cell surface to design and formulate targetable theranostic bioactive agent (BaA) formulations for tumour targeting. This can be achieved by attaching a cancer-targeting ligand to the surface of a bioactive carrier, e.g., a tocosome [3], a liposome, a nanoliposome or other micro- or nano-encapsulation systems [4]. Due to their special characteristics, these carrier systems are able to encapsulate or entrap hydrophilic and hydrophobic agents simultaneously, hence providing a synergistic effect. Since normal cells and healthy tissues do not possess tumour-specific receptors, the bioactive carrier complex precisely targets tumour cells [5]. A main benefit of carrier-based tumour theragnostics is the capability to specifically target tumour cells and hence minimize (or completely remove) adverse effects employing highly explicit targeting ligands, which can accurately bind to target cells and biomarkers [6]. Consequently, carrier-based theragnostics offer a more efficient and less toxic alternative to the conventional therapies. Theragnostic procedures can be utilized to develop strategies for the timely detection and management of different diseases. This is extremely important since timely diagnosis combined with synergistic therapy offered by the theragnostic protocols can significantly improve the chances of successful treatment. Several theragnostic pairs have been suggested and assessed thus far. These include SPECT/PET imaging combined with targeted radionuclide therapy, as well as fluorescent/photoacoustic imaging in combination with chemodynamic/photodynamic/thermodynamic therapy [7,8]. Numerous studies have been performed in order to develop novel theragnostic pairs, including radiolabelled unsymmetric urea derivatives for prostate cancer and synthetic somatostatin analogues for neuroendocrine cancers. The formulation and development of novel theragnostic pairs requires the integration of multidisciplinary procedures, such as novel targeting ligands, novel bioactive carriers and radiochemistry. In addition, conjugation chemistry between parental targeting devices and payloads such as radiometal chelates, radionuclides, cytotoxic agents and fluorescent dyes is also required to achieve optimum outcomes. Fluorescence-based molecular imaging, on the other hand, has become increasingly critical, mainly as a result of the development of very sensitive cameras with enhanced spatial resolutions. These innovations can be utilized, in conjunction with computed tomography, to provide anatomical details of subjects employed for clinical studies.

This Special Issue ‘Theranostic Drug Delivery: Prospects and Problems’ encompasses a collection of research and developments from scientists and practitioners on diverse applications of theragnostic techniques for disease diagnosis and cure. This Special Issue includes 4 high quality review papers and 4 research articles contributed by authors from 14 different countries. The original manuscripts in this Special Issue present novel findings, which will be valuable for the future advancement of theragnostic protocols. In their original manuscript, Roffo and colleagues [9] proposed a one-step microfluidic method (i.e., coupled hydrodynamic flow focusing—cHFF), in order to thermodynamically facilitate the manufacture of complex hybrid nanostructures. These polymer–lipid nanostructures were utilized to co-entrap multiple active compounds for multimodal imaging (i.e., Atto 633 and Gd-DTPA) and theragnostics applications (i.e., Irinotecan and Gd-DTPA). On the other hand, Liu and co-workers [10] reported, for the first time, a novel combinatorial approach of utilizing drug-loaded elastic vesicles following the application of solid microneedle array pretreatment. In another original paper of the special issue, Pérez-Hernández et al. [11] studied multi-sensitive nanocapsule formulations loaded with the antitumour drug T21 both in vitro and in vivo. Results of their study revealed that the amphoteric nanocapsules were internalized more efficiently at slightly acidic pH value (as found in the tumour microenvironment), in comparison with the healthy tissues. The enhanced rate of internalization was associated with a higher level of apoptosis. Furthermore, the biodistribution studies indicated that the nanocapsules were able to target the tumour cells. Their study also revealed that the nanoencapsulation of the T21 drug reduced its systemic toxicity, while maintaining its optimum anti-cancer efficacy. As an added value of their research, and in contrary to other currently available drug nanocarriers, the authors reported that the nanocarriers were prepared by an industrially scalable process using bulk starting materials. Last, but not least, Popova and her research team [12] employed a new labelling methodology for monitoring the spatial biodistribution of the multifunctional gold nanoparticles, as a theragnostic agent, in vivo in real time with high sensitivity and precision. Their formulation was able to reduce the quantity of subjects required for the assessment and development of novel nanoparticles as chemotherapeutic agents and drug candidates for boron-neutron capture therapy. Authors concluded that improving the functional design and development of novel structures for efficient bioactive delivery and targeting are among the essential parameters for optimum theragnostic outcomes.

I hope that the readers of this Special Issue enjoy the broad and innovative content of these articles as much as I did. Taking the quality and novelty of the manuscripts published in this Special Issue, as indicated by being cited more than 160 times (Google Scholar) in such a short period, I sincerely hope that these articles will inspire scientists in their future research and studies.
